# Supplementary material for: Are carbon emissions trading and green financial instruments synergistic? -Comprehensive quantitative research based on content analysis
Source: PLoS One. 2024 Mar 7;19(3):e0298601. doi: 10.1371/journal.pone.0298601 (PMC10919601; doi:10.1371/journal.pone.0298601)
Supplement: S1 Data — (ZIP) [file pone.0298601.s001.zip › Data/地级市Z_F工作报告.zip!/╣π╬≈/╣≡┴╓╩╨/└┤╫╘╣1⁄2╓┌║┼í╛┬φ┐╦╩2╛▌═°í┐_2017.docx]

各位代表：

　　现在，我代表市人民政府向大会作报告，请予审议，并请各位政协委员和其他列席人员提出意见。

　　一、2016年工作回顾

　　2016年是极不平凡的一年，面对错综复杂的宏观环境和持续加大的经济下行压力，我们在自治区党委、政府和市委的正确领导下，按照“加快建设新城，疏解提升老城，产业融合发展，城乡协调推进，生态文化相融，富裕和谐桂林”的总体要求，坚持桂林国际旅游胜地建设“一本蓝图绘到底”，全面推进供给侧结构性改革，全力以赴稳增长、调结构、促改革、惠民生、防风险，全市经济呈现稳中提质、稳中向好的态势，社会保持和谐稳定，实现了“十三五”良好开局。初步预计，地区生产总值完成2074亿元，增长7%;组织财政收入223.8亿元，增长7%;固定资产投资2131亿元，增长16%;社会消费品零售总额831亿元，增长10.5%;城镇居民人均可支配收入30926元，增长7.5%，农村居民人均可支配收入16365元，增长10.4%;居民消费价格指数上涨2.3%;万元地区生产总值能耗下降4.5%，减排指标完成自治区下达任务。

　　——— 胜地建设加快推进。胜地建设发展基础不断加强，体制机制不断创新，旅游与一二三产业融合发展的现代产业体系不断完善。投资300亿元的万达文化旅游城开工建设，国内首家由地级市设立的航空公司——— 桂林航空正式运营，崇华中医街、益田西街建成开业，旅游综合服务功能更加完善。接待游客5380万人次，旅游总消费达626亿元，分别增长20%、21%，旅游综合实力与风向标作用显著增强，世界一流的旅游目的地、全国生态文明建设示范区、全国旅游创新发展先行区、区域性文化旅游中心和国际交流的重要平台加速形成。

　　——— 经济结构持续优化。第三产业增速分别高于第一产业、第二产业4.5、2.5个百分点，对经济增长的贡献率达46%，提升8.7个百分点;高新技术产业增速高于规模工业增速10.7个百分点，经济发展的平衡性、协调性和可持续性进一步增强。

　　——— 发展动能迅速成长。新增市场主体3.95万户;限额以上网上零售额增长785.3%;获批国家重点实验室1个、国家地方联合工程实验室3个，自治区工程技术研究中心2家、重点实验室培育基地5家、企业技术中心7家、技术创新示范企业5家，认定工业新产品44个，数量均居全区前列;与华为、中兴、浪潮等世界500强、国内500强企业成功签约;多渠道融资超300亿元，为经济发展提供了强力支撑。

　　——— 城乡面貌深刻变化。打造品位休闲之都取得重大进展，新区市民广场、山水公园等项目即将建成，市直机关加快入驻，人气、景气、商气日益聚集;“北通南畅、东拓西联”工程基本完成，国道321桂林至阳朔段扩建工程主体基本完工，福利路二期建成通车，循环互通的城市立体交通格局加速形成。荔浦成为国家新型城镇化综合试点县。第二批15个新型城镇化示范乡镇“书记工程”通过验收。“美丽乡村”建设保持全区领先，36个村入选中国传统村落名录，完成首批17个示范村建设，农村人居环境持续改善。

　　——— 改革攻坚取得突破。桂林经开区正式挂牌成立;市级旅游资源整合取得突破性进展，漓江城市段游船实现统一经营;13家市级投融资公司和22家国有企业改革重组为8家市级投融资公司进展顺利;旅游产业用地改革继续走在全国前列，导游自由执业试点中期运行评估全国第一;交通行政综合执法改革试点工作圆满完成。一些多年难啃的改革硬骨头取得突破性进展，为加快发展注入了强劲动力。

　　——— 文化名城魅力彰显。逍遥楼、正阳东巷历史文化街区建成开放，成为代表桂林形象的新名片、新地标。与中国歌剧舞剧院联合创作大型民族歌舞剧《刘三姐》，成功引进乌克兰芭蕾舞剧《天鹅湖》等高雅艺术。央视2016年端午特别节目“歌从漓江来”收视率排名全国同时段第一，国庆黄金周漓江和灵渠旅游在央视连续播出，2017年央视春晚南方分会场花落我市，海内外将再次刮起“桂林旋风”。

　　——— 漓江保护成效显著。漓江流域21家采石场关停拆除复绿;漓江城市段60个截污项目全面完成，城市污水集中处理率提高到97%，非法采沙、住家船、洲岛鱼餐馆、网箱养鱼、畜禽养殖场等清理整顿工作取得阶段性成果，长期困扰漓江保护的难题得到有效化解。漓江城市段沿岸景观亮化工程建成使用;市内游新航线正式开通;沿岸慢行步道改造工程基本完工;精华游游船星级改造全面完成，漓江生态环境持续向好，受到广大市民、游客的普遍好评。

　　——— 社会民生持续改善。全年民生支出311亿元，占一般公共预算支出的77.9%。整合各类社会扶贫资金35亿元;发放扶贫小额信贷12亿元;114个贫困村脱贫摘帽，减少贫困人口7万人，进一步补齐民生短板。落实全民参保计划，新农合参合率达99.01%。荣获“全国法治宣传教育先进城市”，成为广西唯一实现“四连冠”的城市。

　　各位代表，成绩令人鼓舞、催人奋进。这些成绩凝聚着全市人民的辛勤汗水，饱含着社会各界的贡献付出，坚定了我们砥砺前行的决心和信心!

　　过去的一年，困难比预料的多，挑战比预想的大。我们始终坚持以国际旅游胜地建设统揽全局，聚焦目标，迎难而上，重点做了以下工作：

　　(一)注重质量效益，推进产业做强升级。坚持以质量效益为中心，发展壮大主导产业，努力提升传统产业，大力发展高新技术产业、现代服务业和现代农业，现代产业体系不断完善。

　　现代工业稳速提质。产业发展空间进一步拓展，“两翼齐飞、双轮驱动、多点支撑”的工业发展新格局加快形成。桂林经开区建设全面推进，华为产业园等30个项目实现签约开工，邻里中心、大溪河物流园等22个基础设施建设项目初见成效;高新区提质发展，新增高新技术企业10家、发明专利638项、“新三板”上市企业2家，高新区创业园成为广西唯一的A类国家级孵化器，新增在孵企业82家，民华科技入选“国家级科技企业孵化器”，引导成立5家各具特色的民营众创空间，入驻创业团队70个，成为广西大众创业、万众创新的亮点;县域工业园区(集中区)稳步发展，为全市工业发展提供了有力支撑。重大产业项目进展顺利，中电科光电子光通信科技创新与产业化、燕京漓泉100万吨产能填平补齐工程等一批项目开工;溢达九美桥时尚园、桂林电科院电工电子新材料产业基地等一批续建项目顺利推进;华能桂林燃气分布式能源(一期)、广陆数控高端数显量具量仪等一批项目竣工试产。3家企业获自治区“互联网+制造业”示范项目，数量居全区前列;2家企业3款车型进入工信部新能源汽车推广目录。质量品牌建设实现新突破，国际线缆、莱茵生物荣获“中国质量诚信企业”称号，三金药业获自治区主席质量奖，“俏天下”成为中国驰名商标。服务企业成效明显，大力开展“企业直通车”“市领导联系服务重点企业”“干部入企联系服务”等活动，“惠企贷”业务扎实推进。预计规模工业总产值2528亿元，增长7%;规模工业增加值722亿元，增长6.7%，工业经济发展质量效益加快提升。

　　现代服务业优化提升。大力实施“旅游+”战略，推动旅游产业转型升级。重大旅游项目加快建设，桂林国际智慧健康旅游产业园等重点项目进展顺利。旅游“双创”[1]工作全面推进，阳朔、兴安、龙胜、恭城、雁山成为国家全域旅游示范区创建单位，龙胜跻身广西特色旅游名县，雁山、恭城成为广西特色旅游名县创建县，八大生态休闲旅游精品线路[2]加快打造。旅游秩序综合整治深入实施，建立旅游综合监管、联合执法常态化机制，桂林市及阳朔、兴安、龙胜3县旅游巡回法庭相继成立。旅游影响力进一步扩大，成功举办第十届联合国世界旅游组织/亚太旅游协会旅游趋势与展望国际论坛、2016中国—东盟博览会旅游展、第六届桂林国际山水文化旅游节、首届桂林国际马拉松赛等重大活动，被评为2015—2016年度“中国最具魅力会议目的地”和“中国会展名城”。现代服务业加快发展。国家服务业综合改革试点工作被评为全国服务业综合改革十大示范典型之一。服务业百项重点工程建设取得明显成效，漓东中央商务区等20个服务业集聚区建设全面启动。桂林米粉百亿元产业工程加快推进。8家企业获自治区服务业品牌企业称号。“桂林电商谷”入选“全国优秀电子商务园区”20强，拥有电商及关联企业330余家;全州、龙胜、恭城成为2016年“全国电子商务进农村综合示范县”。银行业金融机构存贷款余额分别达2997亿元、1863亿元，增长15%、17.8%，桂林银行完成新一轮增资扩股。预计第三产业增加值781亿元，增长9%，成为稳增长的重要力量。

　　现代农业增产增效。现代特色农业“7+3”提升行动[3]成效初显，粮食总产量达206.7万吨，水果、蔬菜、食用菌对农业总产值增长贡献率超60%，成为全区首个水果业产值超百亿元的地级市。生猪、水产品等效益稳定，南方现代草地畜牧业项目增至9个，数量占全区的一半。现代特色农业示范区加快创建，新增自治区级3个、市级8个、县级21个、乡级27个;确定精品园创建点41个。休闲农业继续走在全国前列，荔浦成为我市第六个“全国休闲农业与乡村旅游示范县”，入选总数占全区一半以上;三星级以上全国休闲农业与乡村旅游示范点增至16个。农产品质量体系逐步完善，阳朔主导制定的《金桔国家标准》颁布实施，“三品一标”使用权认定产品增至117个，荔浦、平乐跻身国家级出口食品农产品质量安全示范区。农业产业化水平不断提高，获国家级、自治区级示范合作社分别为5家、49家，新增市级示范家庭农场13家。农村土地承包经营权确权登记颁证工作稳步推进，广西首家县级林权交易中心在永福成立。农业基础设施进一步夯实，实施中小河流治理项目13个，完成中小型病险水库(闸)除险加固24座，主要农作物耕种收综合机械化水平达52.6%。桂林市第三次全国农业普查工作稳步推进。成功举办第十一届广西名特优农产品交易会，受到自治区领导和与会代表的高度肯定。预计第一产业增加值364亿元，增长4.5%，现代农业发展水平不断提高。

　　(二)注重项目带动，扩大有效投资。坚持每月召开重大项目推进会，实施重大项目动态管理，健全重中之重项目联席会议制度、市领导联系重大项目制度、项目指挥长负责制、县区推进重大项目责任制，项目建设实现新突破。实施重中之重、市领导跟踪联系、自治区和市级统筹推进重大项目787项，预计完成投资748亿元。交通基础设施建设强力推进，两江国际机场T2航站楼主体工程顺利封顶;桂林北动车运用所、桂林西货运中心竣工;资源至兴安、桂林至三江高速公路超额完成年度投资计划，荔浦至玉林高速公路开工建设，灌阳至平乐高速公路完成前期工作年度目标;国道322永福苏桥至鹿寨桂林段完工，全州才湾至资源梅溪、龙脊梯田风景名胜区大循环公路等项目加快推进，立体交通网络不断完善。重大水利基础设施建设进展顺利，斧子口水库实现大坝碾压砼封顶，长塘水库前期工作顺利推进。能源、通信基础设施建设实现大发展，桂北风电基地、桂林南500千伏输变电工程等加快推进，新增风电装机容量25万千瓦。这些重大项目的实施，促进了经济增长，优化了产业结构，提升了城市形象，改善了民计民生，锻炼了干部队伍，有力推动了全市经济社会健康发展。

　　(三)注重统筹协调，提升城乡品质。大力推进中心城市带动、县镇村联动、各种要素互动发展，“城乡一体、生态美丽、文化多元、富裕和谐”大桂林大家园格局加速形成，常住人口城镇化率达48.6%。临桂新区建设再提速。临桂新区与临桂区合署办公。西城大道提升整治工程、创业大厦和世纪立交节点城市设计、防洪排涝及景观水系工程等重点项目基本完成。公共设施日臻完善，道路、桥涵、公交、通讯、水电气等配套项目加快推进;新龙中学等4所学校建成招生，桂林中学临桂校区、桂林师专临桂校区、旅游综合医院等项目进展顺利;一批高端酒店、商业街建成营业或即将投入使用，新区宜居宜业水平进一步提高。老城疏解提升再上新台阶。市区道路整治(一期)工程全面完工，漓江桥扩建、龙门大桥、香江饭店路口立交桥新建、东二环路整修等项目强力推进，桂林始发站站前片区改造竣工，桂林汽车客运南站搬迁工程启动，2座人行天桥建成通行。福隆园、塔山片区等重点棚户区及城中村改造项目加快实施，14条特色街区改造工程全面启动，老城面貌和功能品位持续提升。城市管理水平不断提高。出台实施《城市管理绩效考评办法》。启动城市建筑垃圾公司化处置和“城市双修”[4]工作，成为广西唯一的垃圾分类试点城市。加强市容管理，对119条城市道路进行深度清洗，完成21个非机动车停放点绿化美化;拆除147万平方米违法建筑。新建、改造市政公共厕所48座;完成公共自行车服务系统项目二期、城市地下管线信息管理平台建设，首批100个“爱心驿站”建成使用。出租车整治工作巩固提升。启用数字化城市管理平台，城市管理向常态化、精细化转变。县城及重点乡镇建设日新月异。县城新区建设进展顺利，全州国家新型城镇化综合试点工作初见成效，新增广西新型城镇化示范县6个，数量占全区近1/3。恭城莲花镇入选首批中国特色小镇，阳朔兴坪镇荣获国家投融资创新模式特色镇;广西新型城镇化百镇示范工程增至23个。第三批16个新型城镇化示范乡镇“书记工程”启动建设。“美丽乡村”建设步伐加快。9个广西特色名镇名村完成示范建设任务。建成村际联网公路413公里，“四好农村路”[5]建设和渡口渡船标准化改造成为全区典范。开通城乡公交线路189条，城乡公交一体化“桂林经验”在全区推广。

　　(四)注重改革创新，增强发展活力。着力推进供给侧结构性改革。制定实施国有及国有控股企业重组、“僵尸企业”处置方案，淘汰了一批落后产能;出台支持农民工等新市民购房需求实施细则，大力推动棚户区改造货币化安置，商品房待售面积下降6.9%;出台降低实体经济企业成本40条措施，落实降税清费、阶段性降低社保费率等系列政策;努力补齐产业发展、基础设施、生态环境、社会民生等领域短板。加快推进重点领域改革。重点推进园区体制、投融资、国企国资等一批触及深层次问题、事关我市长远发展的重大改革。安置19家非工业国有企业员工464人。国有林场改革试点工作扎实推进。落实审批事项“接放管服”工作，取消、调整行政审批事项33项，非行政许可审批事项全面取消，在全区率先建立政府权力清单和责任清单动态管理机制。不动产登记办证效率加快提升。全面实施“六证合一”[6]等工商登记制度改革。完成32个乡镇“四所合一”[7]试点改革。全面推开营改增试点工作。城市公立医院综合改革加快推进，药品实现零差率销售，制定分级诊疗实施方案。教育、科技、文化及社会治理等领域改革不断深化。坚持扩大开放合作。积极融入广西“三大定位”，主动参与珠江—西江经济带等多区域合作，加快推进粤桂黔高铁经济带合作试验区(广西园)建设。招商引资实际到位资金602亿元，实际利用外资5亿美元。不断增强科技创新能力。启动“1020”创新驱动计划[8]，全社会科研投入25.8亿元，增长8%，组织实施科技创新计划项目830项。扎实推进自治区级自主创新示范区建设。每万人口发明专利拥有量达6.2件，成为广西首个国家专利质押融资试点示范城市。实施重大人才工程，出台《桂林市高层次人才引进和培养暂行办法》及实施细则，开展第二批漓江学者设岗和选聘活动，推进人才小高地项目建设，培育和引进一批高层次人才。

　　(五)注重品位提升，加强文化建设。大力实施“寻找桂林文化的力量，挖掘桂林文化的价值”工程，推动文化创造性转化和创新性发展。推进历史文化保护利用。首部实体法《桂林石刻保护条例》颁布实施，历史文化资源调查报告评审工作全面完成，历史文化“散珠碎玉”得到系统抢救保护。大力推进王城片区历史文化旅游街区改造提升工程，靖江王城“拆围透墙”工程基本完工;靖江王府及王陵、甑皮岩国家考古遗址公园加快建设，完成靖江王府维修工程一期。第二批文化标识建设进展顺利，一批名人故居正式开放，桂林博物馆、图书馆新馆开馆。成功举办全区纪念红军长征胜利80周年系列活动，湘江战役纪念馆及全州、灌阳红军长征纪念园(馆)建成开放，桂林烈士纪念碑建成。入选第六批自治区级非物质文化遗产代表性项目名录6个。推进公共文化服务体系建设。文化惠民工程扎实推进，公共文化设施场所全部免费开放，新增村级公共服务中心153个，“百姓大舞台”“百姓大讲坛”“漓江之声”“周末大家乐”“精品书画月月展”等公益文化品牌影响力持续提升。推进文化精品创作。桂林渔鼓《梦圆》作为广西唯一入选节目，赴京参加纪念建党95周年优秀曲艺节目展演，杂技《飞天》荣获第六届国际马戏节比赛银奖，《南溪往事》《潮舞荔江》荣获全国优秀作品奖，《抗战桂林》获广西优秀纪录片一等奖。央视春晚桂林分会场筹备工作全面展开。推进文化产业发展。成立全区首家工艺美术创意产业园;组建桂林市文化体育产业投资发展(集团)有限公司，推动文化与绿色制造、旅游、生态、科技、金融、互联网等深度融合发展。文化创意、文化会展、出版印刷等产业加快发展，动漫产业领跑全区。全国文明城市创建工作扎实推进。

　　(六)注重环境治理，提升绿色发展水平。坚持生态立市，以保护漓江为重点，全面加强保护治理，生态环境持续改善，成为第二批国家生态文明先行示范区。漓江保护迈上新台阶。深入推进漓江“三统”改革，建立健全漓江保护协调机制，出台实施漓江风景名胜区破坏生态资源和景观环境违法行为举报奖励办法、采石场发展规划等一批新规;强力开展漓江综合整治“九大专项行动”，漓江城市段排污综合治理成效显著。持续深化环境综合治理。实行最严格的环境保护制度，率先出台“党政同责、一岗双责”管理办法，严厉惩处一批环境违法主体。实行挂牌督办和销号制，按要求完成环保部综合督查、中央第六环保督察组反馈问题的整改。开展“四尘四气”[9]整治，实施防治大气污染“五大专项行动”，砖厂、燃煤小锅炉整治强力推进，淘汰黄标车6269辆，完成储油库、油罐车、加油站油气回收改造，春节期间禁限燃放烟花爆竹取得明显效果，市区环境空气质量持续向好，优良天数达306天，同比增加10天，PM 10、PM 2.5平均浓度值分别下降8.6%、7.8%。全国水生态文明城市建设试点工作持续推进，主要功能区水质达标率继续保持100%;农村集中式饮用水水源保护区划分工作扎实推进，农村生活污水处理设施第三方运行管理在6县区推开。积极开展土壤污染治理与修复。深入开展“生态乡村”建设活动，加快农村环境连片整治，完成农村改厨改厕改圈试点，11县完成“80%的行政村创建生态村”目标任务。持续实施“绿满八桂”造林绿化工程，森林覆盖率达70.92%，银竹老山资源冷杉保护区晋升为国家级自然保护区。

　　(七)注重民生改善，推动社会事业进步。坚持把保障和改善民生作为一切工作的出发点和落脚点，不断增强人民群众获得感。

　　全力推进精准脱贫。建立完善精准扶贫结对帮扶工作机制，全市5.4万名干部结对帮扶8万多户贫困户，实现贫困村驻村帮扶全覆盖。精准实施脱贫攻坚“七个一批”[10]“十大行动”[11]。全面启动“十三五”499个贫困村整村推进项目，贫困村基础设施条件不断改善。实施易地扶贫搬迁工程，惠及贫困户2730户。实施教育精准扶贫，发放资助资金3.75亿元，惠及学生32.7万人次。实施健康精准扶贫，对贫困人口参加新农合的个人缴纳部分补助60%，有效缓解因病致贫问题。实施产业精准扶贫，增强贫困村屯、贫困家庭发展后劲。

　　加快完善社保体系。城乡居民基本医疗保险制度整合工作顺利完成。实现城镇新增就业6.05万人，失业人口再就业1.39万人，就业困难人员实现就业4130人，农村劳动力转移就业新增6.56万人次，城镇登记失业率3.55%。继续实施稳岗补贴政策，惠及职工10.5万人。强化精准救助，发放城乡低保资金4.98亿元。保障居民合理住房需求，开工建设棚户区改造住房20845套，基本建成保障性住房9153套，分配入住保障性住房9702套，完成农村危房改造12870户，均超额完成年度目标任务。

　　社会事业协调发展。坚持教育优先，促进教育公平，学前教育三年毛入园率、九年义务教育巩固率、高中阶段教育毛入学率分别达85%、97.5%、91%;3个城区通过国家义务教育发展基本均衡县认定;新(续)建学校36所，缓解了入学难问题;3所高中通过自治区特色和示范性普通高中评估验收，高考成绩继续保持全区领先;高校集中区建设扎实推进。卫生计生事业稳步发展，中医药事业加快推进，筹建中医医疗联合体;基层医疗卫生工作进一步巩固，秀峰社区卫生服务中心成为广西唯一被评为“全国百强社区卫生服务中心”的基层卫生机构。全面两孩政策有序实施，诚信计生参与率达95.4%。全民健身活动扎实开展。

　　加强和创新社会管理。法治桂林建设持续推进，“七五”普法开局良好。“平安桂林”建设不断深入，社会治安防控体系不断健全。安全生产、食品药品及农产品质量安全监管等工作加快推进，社会大局和谐稳定。

　　一年来，我们坚持以建设人民满意的法治政府、创新政府、廉洁政府和服务型政府为目标，不断提高施政能力和服务水平。始终坚持依法履职，推进依法行政示范点动态管理，努力预防和化解行政争议，积极配合人大行使地方立法权。落实“一站式办公、一条龙服务、阳光下操作、规范化管理”，推进政务服务集中办理，按时办结率达100%，办理提速83.5%，群众评议满意率99%以上。大力推行“互联网+政务服务”，推进政民互动平台、数据交换平台建设，高效整合公共资源交易平台，信息公开及时率、完整率全区领先。重视人大代表、政协委员、民主党派、无党派人士和各人民团体、社会各界人士的意见，促进决策科学化、民主化。积极开展行政效能督查，有效促进工作落实。认真执行市人大及其常委会的决议、决定，自觉接受人大、政协监督，人大代表建议和政协委员提案办复率100%。全面推进公车改革，取消公务用车3734辆。扎实开展“两学一做”学习教育，全面贯彻落实中央八项规定精神和国务院“约法三章”，严格控制“三公”经费，紧扣“六项纪律”，驰而不息纠正“四风”，查处失职渎职人员207人，党风廉洁建设和反腐败工作取得新成效。

　　一年来，国防和后备力量建设得到加强，实现“全国双拥模范城”八连冠，被评为“全国人民防空先进城市”。民族、宗教、外事、侨务、对台工作、人事、审计、监察、统计、物价、编制、接待、机关事务、档案、供销、保密、口岸、气象、水文、测绘、地方志、检验检疫、防震减灾、决策咨询、社会科学、残疾人、公共机构节能等工作取得新进步，中直、区直驻桂林单位取得新成绩，工会、共青团、妇联等群团组织在经济社会发展中发挥了重要作用。

　　各位代表，一年的成绩来之不易。这是市委总揽全局、科学决策的结果，是市人大及其常委会和市政协监督支持的结果，是各级各部门和全市各族人民团结奋斗、顽强拼搏的结果。在此，我代表市人民政府，向全市各族人民，向人大代表、政协委员，向各民主党派、无党派人士、各人民团体和各界人士，向离退休的老领导、老同志，向中央和自治区驻桂林单位，向驻桂林部队和武警官兵，向所有关心支持桂林发展的海内外朋友，致以崇高的敬意和衷心的感谢!

　　我们也清醒地看到，我市经济社会发展还存在不少困难和问题：经济下行压力依然较大，经济增长内生动力不够强，工业生产增速放缓，企业生产经营仍然困难，民间投资增长乏力;资源环境约束趋紧，土地、资金等要素供需矛盾比较突出;县域发展不平衡，开放型经济发展水平不高;深化改革步伐还不够快;城市管理水平有待进一步提高;补齐民生短板和脱贫攻坚任务艰巨，影响社会和谐稳定等因素还不少，等等。面对困难和问题，我们决不回避，将以勇于担当的态度和责任，切实加以解决，绝不辜负全市人民的期望!

　　二、2017年工作总体要求和目标任务

　　今年是实施“十三五”规划承上启下的重要一年，也是实现“两个建成”目标的关键之年。我们将紧紧把握国家建设“一带一路”和桂林国际旅游胜地纳入自治区“双核驱动、三区统筹”[12]核心战略布局等重大机遇，树立强烈的机遇意识和赶超意识，针对我市经济社会发展的瓶颈和薄弱环节，采取积极措施，加大工作力度，推动桂林经济社会发展再上新台阶。

　　今年政府工作的总体要求是：全面贯彻党的十八大和十八届三中、四中、五中、六中全会以及中央经济工作会议精神，深入贯彻习近平总书记系列重要讲话精神，按照“五位一体”总体布局和“四个全面”战略布局，紧紧围绕自治区第十一次党代会、经济工作会议、市第五次党代会和市委五届三次全会重大部署，坚持稳中求进工作总基调，适应把握经济发展新常态，牢固树立和贯彻落实新发展理念，以提高发展质量和效益为中心，以推进供给侧结构性改革为主线，坚持桂林国际旅游胜地建设“一本蓝图绘到底”，围绕“加快建设新城、疏解提升老城、产业融合发展、城乡协调推进、生态文化相融、富裕和谐桂林”的总体要求，协同推进新型工业化、城镇化、信息化、农业现代化和绿色化，促进经济平稳健康发展和社会和谐稳定，为实现“两个建成”目标奠定坚实基础。

　　经济社会发展的主要预期目标是：地区生产总值增长7.5%左右，力争8%;财政收入增长6%，力争6.5%;规模以上工业增加值增长8%，力争8.5%;固定资产投资增长15%，力争16%;社会消费品零售总额增长10.5%，力争11%;节能减排指标控制在自治区下达的目标范围内;城镇居民人均可支配收入增长8.5%，农村居民人均可支配收入增长10%;居民消费价格指数涨幅控制在3%左右;城镇登记失业率控制在4.5%以内，城镇新增就业5.1万人;常住人口城镇化率达50%。

　　为完成上述目标任务，要重点抓好以下工作：

　　(一)狠抓胜地建设，提升全面发展水平

　　全面实现“两个建成”的奋斗目标，必须以《规划纲要》为引领，聚焦第二阶段目标任务，全力推动胜地建设再掀新高潮。

　　加快做好胜地建设中期评估。坚持目标与问题导向，全面总结胜地建设第一阶段工作，发现问题，找出差距，列出清单，完善措施，扎实做好第二阶段工作。

　　进一步厚植发展优势。按照“世界水准、国际一流、国内领先”的要求，强力推动国家赋予桂林的各种试点、试验、示范政策落到实处，力争国家和自治区赋予桂林更多优惠政策，切实把政策优势转化为发展优势。重点把握桂林国际旅游胜地建设纳入全国五大旅游发展重点工程[13]、争创全国健康医疗旅游示范基地等机遇，争取更多项目纳入国家、自治区规划盘子，最大限度释放胜地建设政策红利。

　　加快旅游提档升级。大力发展全域旅游，加快建设大桂林旅游圈，打造桂林旅游升级版，力争旅游接待总人数增长12%，旅游总消费增长15%。实施“旅游+”行动计划，推动山水风光与历史文化、民族风情、健康养生、乡村旅游、红色旅游、休闲体验、购物消费等要素深度融合，大力推进万达文化旅游城、秀峰桃花江片区精品旅游、阳朔瑞盛旅游休闲世界、漓东百里生态旅游示范带、罗山湖体育旅游开发利用(二期)、全州动感天湖度假旅游区、资源八角寨景区提升等项目建设，推动桂林旅游向高端多元休闲度假转型。实施旅游“双创”行动计划。加快创建阳朔、兴安、龙胜、恭城、雁山5个国家全域旅游示范区，继续抓好“广西特色旅游名县”创建提升，持续推进26个特色旅游小镇建设，加快打造八大生态休闲旅游精品线路。实施“领军企业培育”行动计划。推进市级旅游资源和旅游企业优化整合，鼓励市内景区、景点并购联合，支持旅游企业与资本市场对接，加快培育一批旅游旗舰企业。实施“基础设施提升”行动计划。发挥桂林航空作用，完善航空网络，力争开通桂林—雅加达、桂林—黄山等国际国内航线，不断完善游客服务中心等旅游配套设施，加快建设市区一级旅游集散中心和阳朔、兴安、龙胜二级旅游集散中心，打造桂林旅游穿梭巴士线路，实现交通场站、主城区、重要景区间的无缝对接。建立旅游大数据中心，完善旅游网络营销平台，推广应用“电子行程单”、导游公共服务管理平台等信息化监管系统，建立全市统一的定制旅游电子票务管理系统，推动旅游服务提档升级。实施“旅游市场综合整治”行动计划。进一步完善联合执法体制，加强执法能力建设，强化各职能部门监管责任，发挥中介组织、行业协会的作用，重拳打击不合理低价游、景区内乱设收费项目、无资质经营、无资格执业等违法违规行为。办好第十一届联合国世界旅游组织/亚太旅游协会旅游趋势与展望国际论坛、2017中国—东盟博览会旅游展、第七届桂林国际山水文化旅游节等活动，进一步增强桂林的国际影响力。

　　(二)狠抓实体经济，打好产业做强升级攻坚战

　　振兴实体经济是中央作出的重要部署。必须坚持实体优先，大力推进新型工业、现代服务业、现代农业融合发展，不断优化产业结构，提高产业发展质量和效益。

　　重振桂林工业雄风。全面实施“互联网+制造业”战略，加快“两化”融合，培育壮大产业，推动工业迈向中高端，实现规模工业总产值增长9%以上。培育壮大重点园区。桂林经开区要着力在队伍、环境、服务、创新、政策、资源上下功夫，重点完善园区规划、公共服务配套，扎实推进福龙园聚贤路等基础设施建设，建成7万平方米标准厂房;着力加快产业项目建设，开工建设华为、中兴产业园区规划展示中心、产品展示馆、招商服务中心及华为桂林研发培训基地，完成桂林华为科技双创园一期工程;组建成立中兴通讯桂林科技研究院;启动中兴通讯智慧小镇建设;支持华为、中兴办好第一届年度合作伙伴(桂林)招商大会;完成桂林浪潮双创科技园一期建设，确保展示厅投入使用，力争实现工业总产值超700亿元。高新区要依托国家级、自治区级战略性创新平台，打造高层次人才聚集中心、高科技产业孵化中心和先进技术研发基地，不断完善湖塘总部园等专业园区配套基础设施，加快推进中电科、白云电气等重点续建项目建设，力争实现工业总产值超800亿元。培育发展主导产业。发展壮大电子信息、先进装备制造、医药及生物制品、生态食品等主导产业，重点发展新材料、新能源、节能环保等新兴产业;加快建设制造企业数字化车间和智能工厂，发展网络协同制造、大规模个性化定制等智能制造新模式，组织实施智能制造试点示范专项行动和“机器换人”工程，积极推进20家以上规模企业开展“两化”融合项目建设，促进产业转型升级。培育扶持骨干企业。进一步完善领导联系服务企业工作机制，建立“全市重点帮扶企业库”，破解企业生产经营难题，培育一批龙头企业，打造一批“行业小巨人”，加快培育一批“入规”企业。引导企业加大研发投入，加快开发一批工业新产品，争创一批广西名牌产品和中国驰名商标。

　　加快发展现代服务业。围绕“发展提速、比重提高、水平提升”目标，以大企业、大项目为抓手，推动现代服务业集聚发展，力争第三产业增加值增长10%以上。加快建设桂林国际智慧健康旅游产业园、临桂新区物流配送中心等20个现代服务业集聚区，组建粤桂黔高铁经济带合作试验区(广西园)管委会，做好园区规划、征地等前期工作，开工建设灵川传化智能公路港项目。推动生活性服务业提质升级。大力发展健康养生、商贸、运动休闲等生活性服务业，推进桂林米粉百亿元产业工程。着力提升象山万福广场人气，建成叠彩万达广场，加快建设华润万象城等城市综合体，引导购物中心差异化发展。大力发展民宿经济，积极盘活闲置房产资源。开展“健康养老养生小镇”创建活动，打造“漓水青山，养生桂林”城市品牌。推动生产性服务业做大做强。继续抓好会展业发展，加快建设海吉星食尚港等重大项目，推进电子商务、第三方物流、连锁经营等现代流通业发展;加大电商桂林建设力度，开展“桂林特产行销全国”行动，推进线上与线下、城市与农村消费融合发展。加强政府性融资担保体系建设，构建“4321”新型政银担合作关系[14]，破解小微企业融资难题。重点做好规模以上企业培育入库、服务业统计改革、节庆消费促进等工作，力争新增规模以上企业80家。

　　加快建设现代农业强市。继续实施现代特色农业“7+3”提升行动，切实转变农业增长方式，提升农业产业化、标准化水平，实现第一产业增加值增长4%，力争4.5%。实施粮食稳定增产行动，总产量保持在200万吨左右。发展特色效益农业，巩固提升已授牌的现代特色农业示范区品质，大力新建一批自治区、市、县、乡级示范区。建设一批富硒农产品示范基地。深入推进水果标准园创建工作，大力发展“名特优新”水果产业。突出特色养殖、生态养殖、健康养殖，抓好南方现代草地畜牧业、畜禽渔标准化建设、畜禽养殖废物利用等项目。做优休闲观光农业，坚持以旅促农、农旅结合，扎实开展休闲农业与乡村旅游示范县(示范点)创建、“最有魅力休闲乡村”推介、休闲农业星级评定等活动;鼓励各县继续建设一批赏花基地，打造南方地区赏花旅游目的地。大力实施农业品牌战略，建设一批农产品标准化生产基地，提升农产品质量安全水平，加大桂林农业品牌的宣传力度。积极稳妥推进农村土地流转，重点培育年经营收入超10亿元的大型农业龙头企业，引导、扶持、规范一批农民专业合作组织。完善农业发展支撑保障体系，实施山塘水库除险加固、中小河流治理、水土保持综合治理等项目，建设高标准基本农田，力争主要农作物耕种收综合机械化水平提高到57%;强化国家农业科技园区和示范基地建设，深入实施百万农民科技培训工程;加强农产品专业批发市场、冷链物流设施建设，积极培育流通中介;推广“互联网+现代农业”模式，扎实做好特色农产品网络展销、农超对接等促销工作，完善农产品购销体系。重点抓好桂林市第三次全国农业普查工作，精心办好第十三届广西名特优农产品交易会。

　　(三)狠抓项目建设，打好基础设施攻坚战

　　基础设施建设对经济增长具有明显的“乘数效应”。必须把基础设施建设作为重点投入领域，推动投资有效增长，为经济社会持续健康发展奠定良好基础。

　　强力推进重点项目建设。实施项目带动战略，突出抓好交通、能源、水利、信息等基础设施重点项目建设。实施市级统筹推进重点项目850项以上，完成投资790亿元以上，其中重中之重项目10项、市领导跟踪服务推进的重大项目58项、自治区层面统筹推进的重大项目71项。完善现代综合交通运输体系。加快推进两江国际机场扩建工程;桂林北动车运用所、西货运中心建成使用，开工建设桂林北综合客运枢纽(一期);建成桂林至三江、资源至兴安高速公路，加快推进贺州至巴马桂林段、荔浦至玉林高速公路建设，开工建设灌阳至平乐、桂林至柳城等高速公路，积极争取阳朔至鹿寨高速公路复工;完成国道321桂林至阳朔段扩建工程、阳朔火车站连接线及平乐桂江二桥至潮水等项目建设，加快推进永安关经水车至灌阳、资源枫木至兴安界首等公路建设，新开工梅溪至资源、灌阳洞井至灵川潮田等项目;继续做好巴江口船闸改扩能工程前期工作。优化现代能源体系。

　　重点建设以风电、太阳能为代表的电力和配套送出工程，加快建设兴安风电基地及全州、灌阳、恭城、资源等风电项目。加快水利基础设施建设。斧子口水库实现下闸蓄水，加快推进长塘水库建设前期工作。统筹推进信息基础设施建设。以建设国家电信普遍服务试点城市为契机，大力推进“宽带桂林”建设和光纤入户国家标准执行，加快建设中国—东盟信息港桂林数据备份中心。

　　切实提升项目工作水平。不断完善重大项目推进机制，坚持每月召开重大项目推进会，充分发挥市领导联系重大项目的带动作用、项目指挥长的主体作用、市重大办的统筹协调作用、县区部门的要素保障作用，努力推动更多重大项目落地、建成和投产。

　　积极引导民间投资。优化投资政策环境，发挥政府投资引导作用，创新合作模式，降低准入门槛，进一步激活和扩大民间投资。健全政府和社会资本合作(PPP)工作机制，缩短项目周期，降低项目风险，稳定收益预期;加大对民间投资的融资支持，鼓励和引导民间资本进入交通、民生、社会等领域，培育新的投资热点。

　　(四)狠抓精准扶贫，打好脱贫攻坚战

　　脱贫攻坚工作是第一民生工程。必须紧紧围绕“精准扶贫、精准脱贫”这个中心，全面推进脱贫攻坚“七个一批”“十大行动”，确保5.5万以上贫困人口脱贫、100个贫困村摘帽。

　　推进脱贫重点领域攻坚。统筹推进贫困地区道路、水利、网络、农村电网、贫困户危房建设，加快补齐基础设施建设短板，改善贫困村生产生活条件。有序推进易地扶贫搬迁，完成2.2万人以上搬迁任务。推动扶贫产业发展，实施乡村旅游富民工程，扩大贫困村电商网点建设，促进贫困地区农产品增收。

　　加强贫困地区后续发展能力建设。推进教育精准脱贫，加大资金投入，加强贫困地区师资力量，落实贫困户子女从学前教育到高等教育各阶段学生资助政策，实施“农村贫困家庭高校毕业生精准就业帮扶行动”。抓好农村贫困劳动力转移就业脱贫，健全完善农村贫困劳动力职业技能培训、就业创业服务、劳动维权“三位一体”的工作机制，促进就地就近就业、返乡创业。加大健康扶贫力度，建立贫困户健康档案并完善数据库，做好大病保险、医疗救助、商业健康保险等制度衔接工作。加大金融扶持力度，加快发展扶贫小额信贷，提高信贷资金使用效益。加强对已脱贫农户的动态跟踪帮扶，防止返贫。

　　强化脱贫攻坚督查考核。严格执行党政主要领导负总责的脱贫攻坚责任制，层层压实脱贫攻坚责任。完善脱贫攻坚督查工作机制，建立脱贫攻坚工作逐级督查制度，抓好县乡扶贫开发成效考核和实绩考核。抓好扶贫项目资金监管，规范项目管理。加强财政监督检查，健全完善扶贫资金违规使用责任追究制度，保障扶贫资金阳光运行。

　　各位代表!脱贫攻坚事关人民群众的切身利益，我们一定要以决战决胜的勇气，坚决打赢脱贫攻坚这场硬仗，决不让一户贫困群众在全面小康道路上掉队!

　　(五)狠抓城乡建设，提升新型城镇化水平

　　城乡协调发展是全面建成小康社会的必然要求。必须以新型城镇化建设为引领，加快建设现代宜居城市和宜居乡村，统筹推进城乡协调发展。

　　加快打造品位休闲之都。更加注重赋予城市文化内涵，促进功能城市向文化城市转型，加快打造绿色生态、开放创新、活力迸发、管理高效、特色鲜明的现代宜居城市和文化品位之都。临桂新区要进一步丰富现代、时尚、国际文化元素，打造成配套齐全、产业集聚、生态宜居、人文和谐的现代化新城。金融大厦、建设大厦、投资发展商务大厦、桂林日报社传媒中心、广电中心、创业大厦会议中心等投入使用，上半年列入搬迁计划的市直机关、事业单位全部入驻。完善新区规划设计，建设城市规划馆和会展中心，做好机场路以北、兰塘河以南片区控制性详细规划编制。加强基础设施建设，加快推进复兴小学、桂林中学临桂校区、广西师大附外临桂校区、桂林师专临桂校区、旅游综合医院等项目建设，按三级综合医院标准提升桂林医学院二附院;推进枫林农贸市场等一批商贸服务业项目建设，促进居民入住、人气聚集。全力打通“断头路”，完成防洪排涝及景观水系工程，建成开放市民广场、山水公园;建设城市饮用水第二水源，改善新区水质。推动新区与桂林经开区融合发展，规划建设农产品、家具、汽车、工艺品等专业市场。管理和维护好建成设施，营造良好新区建设环境。老城区要进一步完善旅游、生态、休闲等城市功能，丰富历史传统、地域特色等文化元素，提升文化品位。实施风貌改造提升行动。重点推进福隆园、塔山片区等棚户区及城中村改造，加快实施14个特色街区改造提升工程;加强城市深度保洁，重点对城市主要街道两侧、景区、景点和公共活动场所周边建筑进行清洗改造，加大户外广告整治力度;持续抓好小街小巷、集贸市场、城乡结合部、无物业小区、重要节点风貌改造提升;强力开展“两违”清理整治行动，加强源头治理，力争拆除存量违法建筑200万平方米。深入实施“花化、彩化、美化、亮化”工程，完成蚂蟥洲、大洲岛等洲岛自然生态景区建设。实施基础设施提升行动。全面完成万福东路建设、东二环路整修、市区道路整治(二期)、漓江桥扩建、龙门大桥、香江饭店路口立交桥等工程，实施芳香东路、桃花江北路新建工程，加快桂林汽车客运南站搬迁工程建设，新建一批过街人行天桥。实施产业业态提升行动。严格执行老城区房地产限批政策，加快疏散行政办公、工业发展、大型物流、高密度居住等功能，科学规划腾出空间，重点发展品质旅游、互联网经济、楼宇经济等产业。实施城市管理提升行动。积极推进城市管理执法体制改革，配合市人大常委会出台《桂林市城市市容和环境卫生管理条例》，将有关市直部门及驻桂林单位纳入城市管理绩效考评体系;加快建设智慧城市，大力发展智慧交通、绿色交通、综合交通，积极创建国家“公交都市”，完善老城新区间公交线路，加快规划建设快速公交系统，规范出租车行业管理;完善城市标线、标识、标牌。深入开展城市环境综合整治，加强对渣土运输和建筑工地管理，全面推行城市建筑垃圾公司化处置，抓好垃圾分类试点工作，继续开展市区黑臭水体整治。

　　加快培育发展桂林都市圈。深入实施大县城战略，推动县城向城市转型，加快重点城镇扩容提质，加快形成以中心城市为核心、县城为副中心、重点城镇为支撑的大桂林都市圈。加快灵川与中心城区同城化发展步伐，积极推动荔浦、全州撤县改市，推动符合条件的乡撤乡改镇。做好全州、荔浦国家新型城镇化综合试点工作，推进兴安等6个广西新型城镇化示范县建设，大力推动各县老城提升改造和新区建设，加快完善各县城基础设施和服务功能，打造具有浓郁地域风情的“桂北老家”。引导各县结合主体功能区划和区域发展要求，充分发挥自身优势，大力发展特色主导产业，打造一批工业大县、农业强县、旅游名县、生态美县、综合经济强县，加快形成特色鲜明、优势互补、协调联动的县域经济发展格局。完成第三批16个新型城镇化示范乡镇“书记工程”、第二批9个自治区百镇建设示范工程，开工建设第三批7个自治区百镇建设示范工程，建成一批生态优美、设施完善、特色鲜明、宜居宜业的重点特色城镇。

　　加快推进美丽乡村建设。完善乡村规划设计，加强风貌管控，建立健全长效管理机制，全面启动宜居乡村建设，抓好产业富民、服务惠民、基础便民专项活动。加强农村基础设施建设，实现有条件的建制村100%通沥青(水泥)路，力争“四好农村路”建设工作再上新台阶;深入实施农村饮水安全巩固提升、镇镇建有污水处理厂、新一轮农村电网升级改造、自然村(屯)太阳能路灯亮化等工程。加大传统村落保护发展力度，推进灵川“自治区传统村落保护发展示范县”建设，抓好29个中央补助资金传统村落项目，打造一批美丽村庄和乡邻小镇。

　　(六)狠抓改革创新，培育发展新动能

　　改革、开放、创新是发展的活力源泉。必须坚持把改革开放贯穿于经济社会发展各领域，把发展基点放在创新上，在创新中赢得后发优势，为经济社会发展提供持续动力。

　　深化供给侧结构性改革。持续推动“三去一降一补”各项政策的落地实施。严禁产能过剩行业违规新上项目，有序引导“僵尸企业”平稳退出，加快淘汰落后产能。加大去库存力度，确保商品房库存维持在合理水平，继续抓好支持农民工等新市民购房财政补贴、棚户区改造货币化安置等政策落实。实施好差别化信贷政策，拓宽融资领域，优化信贷投向，提高直接融资比重，进一步强化政府债务的监管、评估和预警，防范债务风险。持续实施企业减税费、降成本等措施，有效减轻企业负担。完善基础设施和公共服务体系，加大政策、资金扶持，尽快补齐民生短板。

　　深化重点领域改革。继续深化行政审批制度改革，抓好“接放管服”各项工作，确保简政放权落到实处。筹备组建行政审批局，推动集中审批。完成城市管理综合执法体制改革。大力推动工商登记便利化改革，完成个体工商户“两证合一”[15]登记。深入推进税制、税收征管体制改革，巩固扩大营改增成果。加大桂林经开区改革力度，建立健全精简高效的园区管理体制机制。继续深化国资国企改革，优化资本配置，完善治理机构，改革监管方式，促进国有资产保值增值。深入推进投融资体制改革，抓好8家市级投融资公司组建工作，推动国资国企在资本市场直接融资。全面完成不动产登记制度改革。切实做好农村土地承包经营权确权登记颁证、农村金融、水利、林业、供销等农业农村改革。积极稳妥推进公立医院综合改革，坚持医疗、医药、医保“三医联动”，推进分级诊疗、现代医院管理、医保支付等制度改革，减轻群众看病负担。继续深化教育、科技和社会治理等领域改革。

　　拓展多领域开放合作。深化面向东盟、对接港澳台、拓展欧美日韩的开放合作，积极参与“一带一路”建设，加强与高铁沿线、珠江—西江经济带、北部湾、珠三角等区域合作。持续推进招大引强，以桂林经开区、高新区等重点产业园区为主战场，围绕四大主导产业、战略性新兴产业、现代服务业、“互联网+”行动计划等开展精准招商、专题招商、产业链招商，重点引进一批世界500强、国内500强和行业领军企业，实现招商引资实际到位资金超640亿元，实际利用外资5亿美元以上。鼓励企业“走出去”，支持有实力的企业开展重大项目国际合作和工程承包。稳定传统优势产品出口，推动农产品出口东盟;加快技术转让、研发设计等服务贸易出口，大力发展跨境电子商务，加强口岸经济建设，力争外贸进出口总额超过58亿元。

　　提升创新发展能力。加快实施“1020”创新驱动计划，积极创建自治区级自主创新示范区。以优势产业和战略性新兴产业为重点，整合各类创新资源，采取“一个产品、一个团队、一家科研机构、一家企业、一支基金”的模式，集中力量开展科技攻关，加速推动科技成果产业化。加快建设一批技术中心和研发中心，力争新增自治区级创新平台10家以上，重点支持桂林电科院、曙光院、电科大、莱茵公司等在相关领域创建国家级创新平台。继续抓好专利倍增计划，力争申请发明专利5300件以上，每万人口发明专利拥有量6.5件以上。加强质量品牌建设，加大自主品牌知识产权保护力度。激发人才创新创造活力，设立人才发展专项资金，在津贴、购房补贴、科研扶持、子女入学等方面，为来桂林干事创业的高层次人才提供全方位支持;以实施漓江学者制度、建设人才小高地、创建人才工作示范点等重大人才工程项目为抓手，继续培育和引进一批高层次创新创业人才。

　　(七)狠抓文化建设，提升城市软实力

　　历史是城市之根，文化是城市之魂。必须坚定文化自信，加大文化作为，加快文化创新，促进文化繁荣，让文化软实力成为桂林经济社会发展硬支撑。

　　加强历史文化保护利用。按照“使文化遗产拥有尊严，成为促进经济社会发展的积极力量，成果惠及民生、让民众有参与感”的思路，深入实施“寻找桂林文化的力量，挖掘桂林文化的价值”工程，“唱”好历史文化保护利用“三部曲”，努力把“散珠碎玉”式的历史文化串联起来，推出一批承载城市记忆、传承优秀文化的研究成果和文化产品，完成《桂林历史文化大典》年度编撰任务。继续做好文物遗址保护修复。加快推进王城片区历史文化旅游街区改造，实施靖江王城城门楼复建工程，完成靖江王府维修工程二期和正阳西巷改造;启动甑皮岩国家考古遗址公园二期工程，抓好靖江王陵保护区散葬民坟治理;继续开展名人故居修缮开放，完成第二批、启动第三批文化标识建设;加快实施灵渠(南渠)二期、古桂柳运河工程、灵川江头村和长岗岭村古建筑群修缮二期、桂林石刻等文物维修工程，持续推进灵渠申遗，办好“国际博物馆日”广西分会场活动。着力打造以历史文化街区、博物馆、历史名人故居等为主线的历史文化旅游线路，继续推进红军突破湘江战役旧址建设。加强非物质文化遗产保护传承和展示工作，开工建设桂林市非物质文化遗产展示馆。加强对民俗文化的挖掘保护和利用。

　　提升公共文化服务水平。深入实施文化惠民工程。持续推进图书馆、文化馆(站)、村级公共服务中心、农家书屋等文化设施建设，抓好宽带网络、广播电视无线覆盖。继续推进“书香桂林”工程。开展“读书月”活动，推进公共场所图书吧、图书角、图书室建设。努力打造文化惠民优秀品牌。积极开展“深入生活，扎根人民”送文化送欢乐下基层、传统文化进校园活动，持续提升“百姓大舞台”“百姓大讲坛”“漓江之声”“精品书画月月展”等群众性特色文化品牌影响力。全力办好2017年央视春晚桂林分会场演出直播。

　　加快文化产业发展。推进文化产业结构优化升级。重点发展文化创意、出版发行、演艺娱乐、文化会展、广播影视等产业，进一步提升力港动漫、可可小爱等品牌影响力。加快高新区文化创意产业园、希宇文化创意产业园、五通农民画产业园、桂林袭汇国际文化世界等项目建设，创建新的国家级文化产业示范园。做足“文化+”文章。推进桂林文化和科技融合示范基地建设，加快文化与旅游、生态、金融、互联网等融合发展，打造一批生态文化相融的景区、景点。积极扩大文化消费。培育新型演出市场，打造阳朔《漓江千古情》等一批知名演艺品牌，做好《藤缠树》桂林大剧院驻场演出，举办中国歌剧舞剧院演出季，打造大型民族歌舞剧《刘三姐》。积极培育文化市场主体，加快文化企事业单位公司制、股份制改造，完成桂林市文化体育产业投资发展(集团)有限公司组建。

　　(八)狠抓生态建设，推进绿色发展

　　良好的自然生态是永续发展的基础。必须牢固树立“绿水青山就是金山银山”的理念，坚持生态立市，持续强化环保目标责任落实，全面改善环境质量，确保桂林天更蓝、地更绿、水更清、空气更清新。

　　全力推进漓江生态保护。按照“保护、利用、管理”原则，坚持“统一经营、统一管理、统筹各方利益”，充分发挥漓江保护协调机制作用，做到发展与绿色同行。严格依法依规保护漓江。认真执行《广西壮族自治区漓江流域生态环境保护条例》《桂林漓江风景名胜区总体规划》，组织编制漓江风景名胜区详细规划，加强漓江综合执法能力建设，建立健全四级网格化责任监管体系，坚决遏制乱建、乱挖、乱养、乱经营等“四乱”现象。强化漓江综合治理。统筹推进漓江风景名胜区生态环境综合整治十项重点工作，突出抓好漓江城市段截污工程(二期)，加快推进漓江流域畜禽禁限养、网箱清理整治等工作。加大漓江上游水源林区及沿岸植被保护建设力度，加强沿江乡镇村屯污水综合治理。加强漓江景区改造提升。完成“两江四湖”二期工程，扎实推进漓江旅游专用码头、漓江城市段游船提升改造、漓江西岸桂阳公路慢行绿道、漓江核心景区沿江可视范围生态景观林等项目建设。创新漓江旅游经营机制，推进漓江分段游、分时游、分区域游，维护漓江旅游秩序，实现综合效益最大化。

　　加强生态环境保护。推进国家生态文明先行示范区和国家森林城市建设，持续抓好中央环保督察组反馈问题的整改工作。强化大气污染防治。继续实施“PM10+PM2.5”双目标联控，深化工业废气治理，实施砖厂气体排放在线监控，持续加强机动车尾气和城市扬尘污染治理，力争优良天数继续保持在300天以上。强化水污染防治。扎实开展全国水生态文明城市建设试点工作，实现河流水国控、区控监测断面水质优良比例达到100%;加强饮用水源地保护和水环境综合治理，加大备用水源地青狮潭水库环境综合整治力度，确保水库水质保持Ⅲ类水平。加强退化土壤修复与改良。完成土壤环境质量国控监测点位设置，开展土壤污染调查，加强农业可耕地土壤治理。加强环境监管和执法力度，严厉查处各类生态环境违法行为，确保环境安全。

　　大力推进生态城乡建设。全面落实主体功能区制度和生态红线区域保护规划，坚守耕地保护、开发强度、生态保护“三条红线”，促进生产空间集约高效、生活空间宜居适度、生态空间山清水秀。推进会仙、荔江、龙脊梯田、灌江、天湖等国家湿地公园及琴潭千亩荷塘湿地公园建设。继续实施“绿满八桂”造林绿化工程，森林覆盖率达70.92%以上。着力改善农村人居环境。深入实施农村垃圾治理两年攻坚行动，重点推进50个乡镇片区垃圾处理中心、113个村级垃圾处理设施项目建设，力争90%以上的村屯垃圾实现有效处理。继续探索实施农村生活污水处理设施第三方运行管理机制，促进污水、垃圾处理等设施建设运营产业化。深入实施农村改厨改厕改圈工作，加快农村环境连片整治。

　　加快发展生态经济。推进清洁生产，发展循环经济，重点推进雁山低碳经济产业示范园区等一批示范园建设，开展绿色制造示范试点，推广绿色制造;大力发展无公害、绿色有机农产品，打造桂林特色生态农业品牌;积极培育优质特色经济林，加快发展林下经济;倡导绿色低碳消费，全面开展服务业节能减碳行动，打造休闲旅游、健康养生、商贸物流等服务业低碳示范区，将生态优势转化为经济优势。

　　(九)狠抓民生改善，增强群众幸福感

　　让人民群众共享改革发展成果，是一切工作的出发点和落脚点。必须坚持普惠性、保基本、均等化、可持续的方向，切实保障和改善民生，让人民群众过上更加幸福美好的生活。力争全年民生类公共财政支出增长10%以上。

　　切实抓好就业创业和社会保障。加强创新创业平台建设，重点依托高新区、桂林经开区等平台，培育构建一批众创空间、众创社区，多渠道增加就业岗位，实现城镇失业人口再就业4.5万人以上、农村劳动力转移就业5.5万人次。巩固扩大各类保险覆盖面，进一步落实全民参保计划，全面提升政策“兜底”水平。完善各类社会救助制度，加强基层社会救助能力建设。推进社会福利慈善事业发展，加快养老服务市场化、产业化步伐。

　　协调发展社会各项事业。继续加大教育投入，新(续)建中小学校47所，进一步解决入学难问题;加快普及三年学前教育，实现乡镇公办幼儿园全覆盖;推进义务教育均衡发展，加强师资队伍配备和培训工作，实施公办初中质量提升计划，补齐教育短板;推动普通高中特色化发展，整合中职教育资源，完成桂林市卫校雁山校区一期工程建设，筹建桂林职业技术学院，推动桂林师专“专升本”;支持驻桂林高校发展，推进桂林高水平大学集聚区建设，引导高校服务地方经济社会发展。全面推进健康桂林建设，完善公共卫生服务体系，强化基层医疗服务能力建设，加强重大疾病防控;持续推进中医药事业发展;加快发展健康产业，推动医养结合，促进医疗与旅游融合;提高计划生育服务管理水平，有序实施全面两孩政策，巩固提升诚信计生工作，人口自然增长率控制在9‰以内。开创全民体育运动新局面，重点办好第二届桂林国际马拉松赛、环广西公路自行车世界巡回赛(桂林站)、中国·阳朔国际山地越野赛等赛事。

　　营造和谐稳定社会环境。加快法治桂林建设，深入实施“七五”普法。深化平安桂林建设，推进社会治安网格化管理，完善突发事件应急处置机制，做好信访维稳和矛盾化解工作。完善公共安全体系，加强食品药品安全监管，争创广西食品安全城市，抓好安全生产、防灾减灾工作，坚决遏制重特大安全事故发生。继续抓好“扫黄打非”工作，加强对吸毒人员的管理教育。贯彻落实党的民族、宗教、外事、侨务和对台政策，促进民族团结进步。加强国防动员和后备力量建设，深入开展双拥共建，推动军民融合深度发展。深化人民防空改革，增强城市综合防护能力。继续推进全国文明城市创建工作。

　　继续实施为民办实事工程。坚持民生优先，筹集资金65亿元，在确保完成自治区下达我市重点民生项目任务的基础上，重点实施市级层面十大类34个为民办实事项目。

　　(十)狠抓政府自身建设，不断提升行政效能

　　面对加快“两个建成”的新机遇、新要求，政府使命在肩、责任重大。必须以提升能力为导向，进一步加强政府自身建设，全面履行好政府职能。

　　建设法治政府。坚持依法行政、依法履职，把政府工作全面纳入法治轨道，推进政府权限、程序、责任法定化。深化行政执法体制改革，严格规范公正文明执法，全面落实行政执法责任制。完善“三重一大”决策程序，坚持执行重大行政决策集体讨论的民主决策制度。建立健全重大决策第三方评估机制，大力推进科学决策、民主决策、依法决策。

　　提高行政效能。推进“互联网+政务服务”，实现实体政务大厅与网上政务服务平台融合发展。健全政务信息资源目录，建立完善公共资源交易平台、政务数据共享交换平台，加快推进部门间信息互认共享与业务协同，真正做到“信息多跑路，群众少跑腿”，最大限度提高办事效率。强化行政审批事中事后监管，努力做到审批环节少、办事效率高、服务质量好。

　　强化权力监督。积极推进督查和审计情况公开、决策执行情况公开、行政审批中介服务事项公开，确保权力公开透明运行。自觉接受人大及其常委会的法律监督、工作监督，政协的民主监督，社会的舆论监督，加大人大代表建议、政协委员提案的督办力度。加强政府督查，对责任心缺失、不作为、乱作为的政府部门及工作人员严格执纪问责，以责任追究倒逼责任落实，确保各项重大决策部署落地生根、早见实效。

　　持续改进政风。巩固“两学一做”学习教育成果，推进作风建设常态化、长效化。加强干部队伍建设，强化政风政纪督查，着力打造一支信念坚定、为民服务、勤政务实、敢于担当、清正廉洁的高素质干部队伍。贯彻中央八项规定精神，严守“约法三章”底线，加强政府系统党风廉洁建设，强化主体责任意识，筑牢拒腐防变的思想防线和制度防线，坚持有腐必反、有贪必肃，切实优化发展环境。

　　各位代表!劳动创造幸福生活，奋斗赢得美好未来。让我们紧密团结在以习近平同志为核心的党中央周围，在自治区党委、政府和市委的正确领导下，保持定力，不忘初心，开拓创新，务实进取，加快实现“两个建成”，以优异成绩迎接党的十九大胜利召开!
